# Brownian dynamics study of the association between the 70S ribosome and elongation factor G

**DOI:** 10.1002/bip.21619

**Published:** 2011-09

**Authors:** Maciej Długosz, Gary A Huber, J Andrew McCammon, Joanna Trylska

**Affiliations:** 1 Interdisciplinary Centre for Mathematical and Computational Modeling, University of WarsawWarsaw, Poland; 2 Howard Hughes Medical Institute, University of California San DiegoLa Jolla, CA 92093-0365; 3 Department of Chemistry and Biochemistry, University of California San DiegoLa Jolla, CA 92093-0365; 4 Department of Pharmacology, University of California San DiegoLa Jolla, CA 92093-0365; 5 Center for Theoretical and Biological Physics, University of California San DiegoLa Jolla, CA 92093-0365

**Keywords:** Brownian dynamics, 70S ribosome, translation

## Abstract

Protein synthesis on the ribosome involves a number of external protein factors that bind at its functional sites. One key factor is the elongation factor G (EF-G) that facilitates the translocation of transfer RNAs between their binding sites, as well as advancement of the messenger RNA by one codon. The details of the EF-G/ribosome diffusional encounter and EF-G association pathway still remain unanswered. Here, we applied Brownian dynamics methodology to study bimolecular association in the bacterial EF-G/70S ribosome system. We estimated the EF-G association rate constants at 150 and 300 mM monovalent ionic strengths and obtained reasonable agreement with kinetic experiments. We have also elucidated the details of EF-G/ribosome association paths and found that positioning of the L11 protein of the large ribosomal subunit is likely crucial for EF-G entry to its binding site. © 2011 Wiley Periodicals, Inc. Biopolymers 95: 616–627, 2011.

## INTRODUCTION

The ribosome, protein synthesis machinery, is a ribonucleoprotein complex composed of two subunits that in bacteria are named 30S and 50S based on their sedimentation coefficients. These two subunits, which consist of three RNA chains and over 50 proteins, interact through a network of intersubunit bridges to form the 70S active ≍2.5 MDa ribosome. Bacterial translation is a complex and multistage process that requires the formation of peptide bonds according to the sequence residing in the mRNA. Amino acids necessary for peptide synthesis are covalently bound to CCA-termini of transfer RNAs (tRNAs). There are three tRNA binding sites on the ribosome located at the interface between subunits: A, aminoacyl; P, peptidyl; and E, exit. The A-site accepts the incoming aminoacylated tRNA, the P-site holds the tRNA with the growing peptide chain, and the E-site interacts with the deacylated tRNA before it dissociates from the ribosome. Peptide bond synthesis takes place between the aminoacyl and peptidyl termini of the A- and P-site tRNAs. After the reaction, tRNAs translocate to their new positions (P and E) with the advance of mRNA by a distance of one codon to preserve the reading frame. Next, the peptide elongation cycle is repeated and cognate-tRNAs bind to the A-site until a stop-codon on the mRNA is recognized.

The stages of bacterial translation are accelerated by external protein factors that bind to the ribosome. The key factors include initiation factors, IF-1, IF-2, and IF-3, which assist in subunit association, elongation factors EF-Tu and elongation factor G (EF-G) that catalyze the peptide elongation stage, and release factor of the termination stage. In the elongation stage, EF-Tu-GTP complexed with the aminoacyl-tRNA delivers the tRNA to the ribosomal A-site. The recognition of a cognate charged tRNA results in its accommodation in the A-site, GTP hydrolysis, and further release of the EF-Tu-GDP complex. Subsequently, the ribosome catalyzes the peptide bond formation between the amino acids bound to the CCA-termini of A- and P-site tRNAs. In the next stage, EF-G-GTP associates and, also at the expense of GTP,^1^ accelerates the translocation of tRNAs and mRNA. Next, EF-G is released and the ribosome in its post-translocational state is ready to accommodate another cognate tRNA.

All these steps, as each amino acid is added to the growing peptide chain, involve various structural rearrangements of the ribosome and external factors. Overall, the ribosome is a dynamic macromolecular complex and its large-scale fluctuations are necessary for protein synthesis. The internal motions of the whole 70S ribosome have been probed by X-ray crystallography,^2–5^ cryo-electron microscopy (cryo-EM),^6–9^ and single molecule FRET experiments,^10–14^ as well as simulations.^15–19^ The studies of the dynamics of the ribosome have been reviewed by many authors, for example, Refs. 20–28. The lowest frequency global motion observed by cryo-EM was the ratchet-like rotation of the subunits.^7^ Apart from the subunit rotation required for translocation of tRNAs, the ribosome collective motions also involve the movement of the 50S subunit stalks L1 and L7/L12 together with the L11 protein, the swivel and tilt of the head of the 30S subunit relative to its body, and numerous local rearrangements, for example, in the decoding center. The ribosome atomic structure has about 750,000 degrees of freedom and thus can access multiple conformations. Naturally, the ribosome has an intrinsic capacity to sample an ensemble of states. The external factors help the ribosome acquire a certain conformation from this ensemble^24^ and direct the path to another state.

**FIGURE 1:**
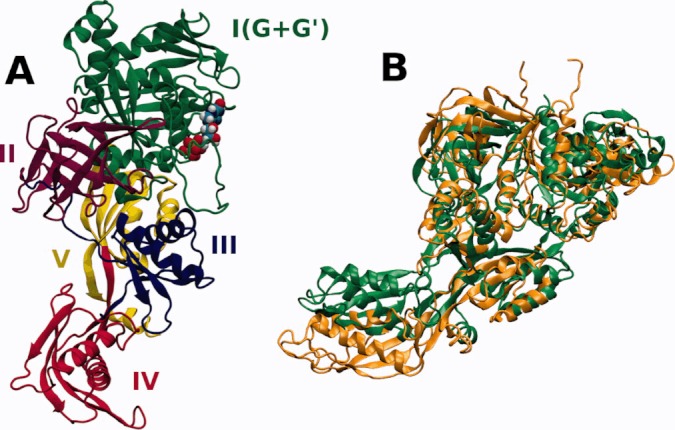
(A) A cartoon model of the EF-G protein from *E. coli* (PDB code 1FNM^29^), colored according to the tertiary structure domains. The GTP molecule with bound magnesium ion is shown as spheres. (B) Two superimposed structures of the EF-G protein, isolated (green, 1FNM^29^) and trapped on the 70S ribosome in the post-translocational state (orange, 2WRI^5^).

**FIGURE 2:**
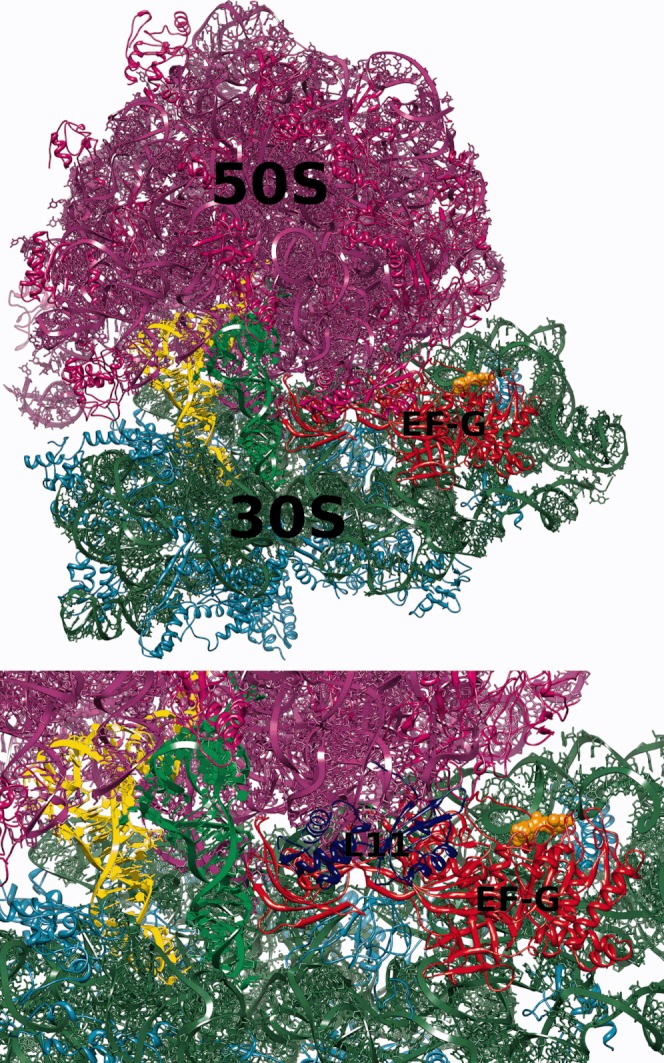
Top: A model of the 70S ribosome/EF-G complex used in this work. The 50S subunit-proteins are shown in magenta, RNA in violet; 30S subunit-proteins are in cyan, RNA in green; EF-G is in red, GTP is shown as orange spheres, A-site and P-site tRNAs are in green and yellow, respectively. Bottom: A detailed view of the A-site with the ribosomal protein L11 shown in blue.

Here, we focus on the process of association of EF-G with the 70S ribosome. EF-G is a five domain protein composed of over 600 amino acids (see Figure 1). It is of elongated shape with domains III, IV, and V mimicking the shape of the tRNA molecule.^30–35^ Domains I (or G) and II form a unique β-barrel. The domain I of EF-G includes an insertion—the G′ subdomain. EF-G is a GTP-driven factor whose overall charge at pH 7 in solution is −22*e* (including GTP and magnesium ion). EF-G binds at the interface between ribosomal subunits in an elongated cleft close to the L11 protein (see Figure 2). Even though both molecules are negatively charged (ribosome net charge without counter-ions is about −4500*e*), there exists some electrostatic complementarity between EF-G and its binding site on the ribosome.^36^

The crystallographic studies on EF-G have shown that the EF-G conformation in the crystal does not depend on the type of the ligand bound GTP or GDP.^29^, ^34^, ^35^, ^37^ Nevertheless, EF-G in solution due to its elongated shape and multidomain composition is a flexible protein^38^, ^39^ whose domain IV has an inherent property to bend.^39^ The domain IV of EF-G is crucial for rapid translocation and EF-G release from the ribosome.^1^ The conformation of EF-G elongated part changes upon binding to the ribosome in comparison with its unbound or crystal conformation. The fitting of the EF-G crystal conformations to the cryo-EM density maps of EF-G as bound to the ribosome have confirmed these EF-G conformational changes at atomic detail.^15^, ^40^, ^41^

Also, EF-G while bound to the ribosome retains its dynamics.^5^, ^8^, ^42^ For example, the motions of the G′ domain toward the N-terminal domain of the L11 protein are believed to be important for translocation.^10^ However, the details of how the EF-G acts on the ribosome and how the ribosome movement is coupled to EF-G movement are still to be elucidated. Also, most of the structural studies visualize EF-G as bound to the ribosome and are not able to characterize the initial stages of binding, that is, the association paths of EF-G diffusing toward the ribosome and if and how EF-G is directed to the binding site.

The association rate constant of EF-G-GTP with the *E. coli* ribosome was determined experimentally as 1.2–1.5 × 10^8^ M^−1^ s^−1^ and the rate of EF-G-GDP as 9 × 10^7^ M^−1^ s^−1^.^1^ The experiments were performed at pH 7.5 and (50 m*M* Tris-HCl) + (30 m*M* KCl) + (70 m*M* NH_4_Cl) + (7 m*M* MgCl_2_). The association rate constant was found to be roughly independent on the ligand: GTP or GDP. For typical protein–ligand association, the diffusion limit is generally taken as 10^8^ M^−1^ s^−1^.^43^ Second-order rate constants for protein–protein association can be either lower, of ∼ 10^6^ M^−1^ s^−1^^44^ or higher, of the order of 10^10^ M^−1^ s^−1^,^45^, ^46^ when electrostatic interactions involving opposite charges on protein surfaces or significant electrostatic steering come into play. Therefore, the catalytic rates of EF-G seem to be rather rapid, especially considering the fact that both EF-G and the ribosome are of negative charge.

Here, we applied Brownian dynamics (BD) methodology to investigate the first stages of binding of EF-G with the ribosome and to determine its association rate constants. BD is a powerful method to simulate the diffusional motion between interacting molecules^47–49^ and has been commonly used to theoretically estimate association rates of diffusion controlled bimolecular reactions. In the BD framework, the two interacting solutes move in a continuum solvent that exerts stochastic forces inducing their random motion. Intermolecular electrostatic interactions are described with the Poisson-Boltzmann model.^50^ On the basis of the number of association events (encounters) observed in many BD simulations, one estimates the rates of bimolecular association.^51^, ^52^ BD has been previously applied to study protein–protein,^53^, ^54^ protein–ligand,^55^ and DNA/RNA–ligand interactions.^56–58^ In this work, we apply BD to a much larger ribonucleoprotein complex. Because the ribosome alone contains over 250,000 atoms, BD studies pose a challenge not only with respect to computer time and resources, but also analysis.

The aim of this study is to characterize the most probable pathways of association of the EF-G factor with the 70S ribosome in its pretranslocational state. Additionally, we provide a theoretical estimation of EF-G association rate constants at two ionic strengths. We analyze if EF-G is directed toward the ribosomal binding site and how electrostatics influences the association.

## RESULTS

### Association Pathways

**FIGURE 3:**
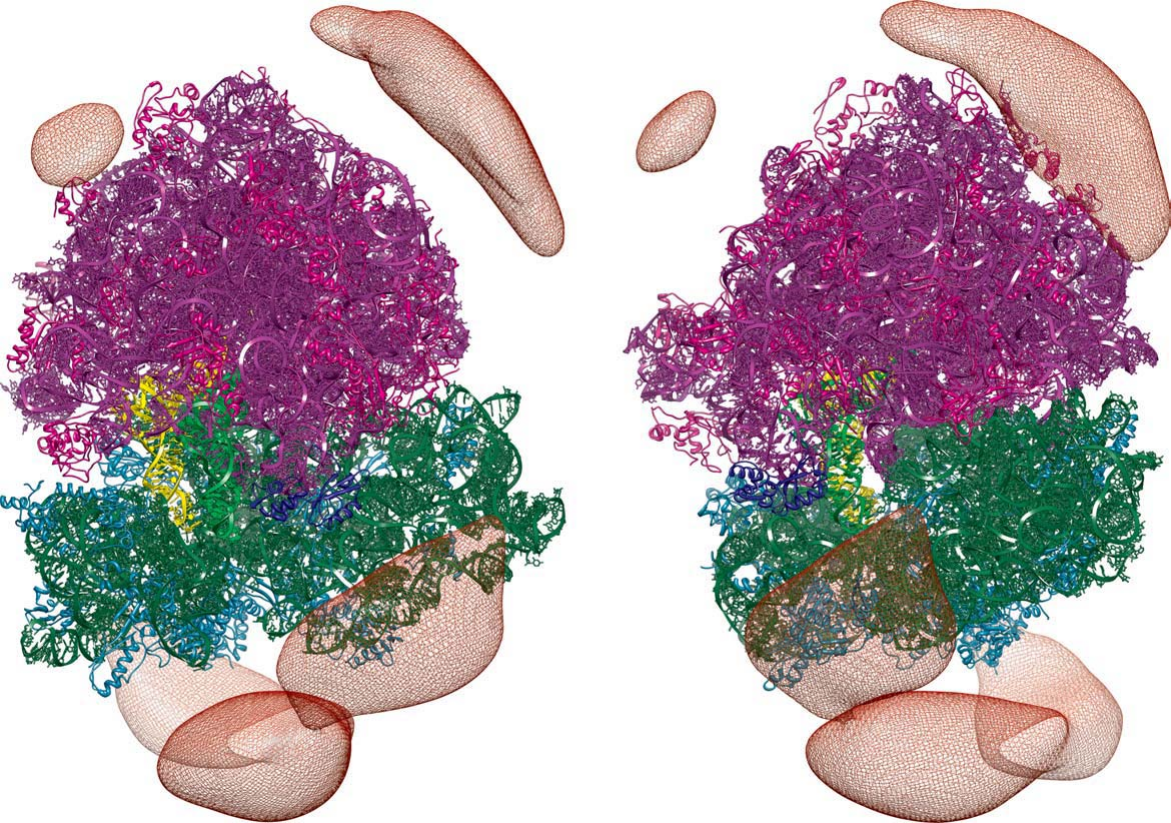
Constant density surface (red mesh) of EF-G around the 70S ribosome shown for two different ribosome orientations. The ribosome coloring as in Figure 2. The probability of finding EF-G inside the depicted surface is ∼ 20%.

**FIGURE 4:**
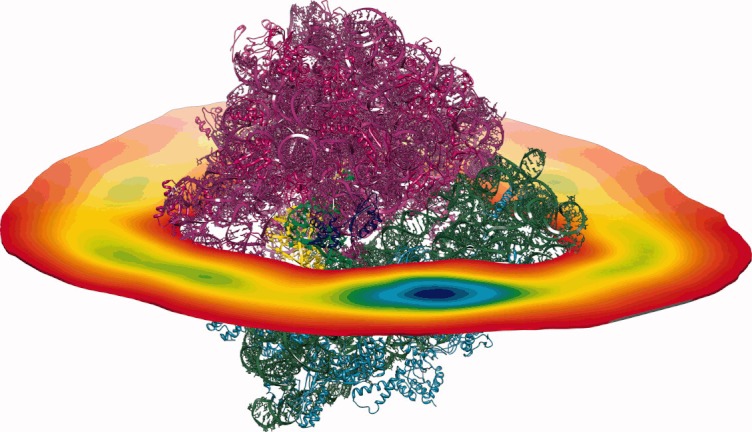
A slice through the density map of EF-G around the 70S ribosome. Colors change from red (low density) to blue (high density). The ribosome coloring as in Figure 2.

Initially, 1,000,000 BD trajectories of EF-G diffusing in the electrostatic field of the 70S ribosome were generated. These simulations were conducted at 150 m*M* of monovalent ionic strength. Here, the reaction criteria used to determine whether the encounter complex was formed were defined based on the distances between the atoms of EF-G and the ribosome observed in the complex (see Figure 2 and the Materials and Methods section). For that, a list of distinct pairs of EF-G and ribosome atoms within a distance in the complex below 5 Å was determined. At first, we assumed that the encounter complex was formed if the distance within any two of chosen pairs observed during simulation was less than 10 Å. However, this definition turned out to be too restrictive (the number of successful trajectories out of 1000,000 was only 92, giving a rate constant 5 × 10^6^ M^−1^ s^−1^ that is lower than the value obtained experimentally). Moreover, in all registered encounter 70S ribosome/EF-G complexes, the EF-G protein was located far from the ribosomal A-site with the domain IV pointing outside the ribosome. EF-G was rotated relative to its position in the complex and interatomic contacts observed in encounter complexes did not involve atoms of the domain IV. The domain IV is the flexible part of EF-G^39^, ^40^, ^59^ mimicking the anticodon arm of the tRNA molecule,^30–35^ and its final positioning in the EF-G/ribosome complex is toward the A-site.^5^ However, such tight reaction criteria enabled us the detailed analysis of the possible association paths and observation of the most probable sites that EF-G occupies around the ribosome and in the vicinity of its binding site. Using 50,000 randomly chosen BD trajectories from the generated set of 1,000,000, we next constructed the EF-G density maps, as described in the Materials and Methods section, to visualize EF-G preferred positions around the ribosome (Figures 3 and 4).

We used only a subset (50,000) of all generated trajectories (1,000,000) as the preparation of density maps involves rather significant number of arithmetic operations performed on large data sets. Constant density surfaces presented in Figure 3 suggest that there is no electrostatic steering that would direct EF-G toward its binding site on the ribosome. Although a preferred region with a significant volume is clearly visible near the L11 protein, there are also other regions on the ribosome surface that are visited frequently by the factor G—especially those that are rich in proteins. For example, such regions are visible in Figure 3, around proteins L4, L15, L23, L24, and L29 (of the large subunit) and S2, S3, S4, and S10 (of the small subunit). Although in the employed ribosome structure EF-G is not able to efficiently penetrate the elongated and tight pocket near the ribosomal A-site, in Figure 4 a blue spot denoting a significant EF-G density is present near the binding pocket, in the vicinity of the L11 ribosomal protein (see also Figure 2). Following the density maps we identified the blue spot of Figure 3 as the position of the initial 70S ribosome/EF-G encounter complex. The density analysis enabled us to better define the reaction criteria in BD simulations and use them to estimate EF-G association rates.

### Association Rates

**FIGURE 5:**
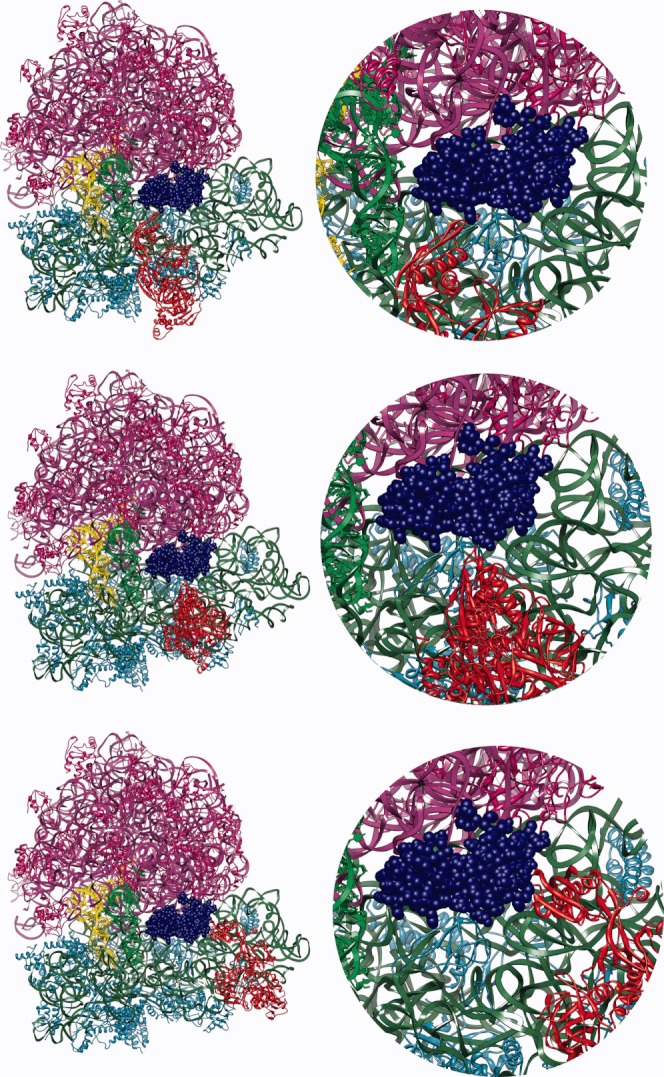
Encounter complexes observed during BD simulations. The ribosome and EF-G coloring as in Figure 2.

Further BD simulations were set up and performed in order to estimate the rates of EF-G association. For this purpose, we defined the reaction criteria based on the previous density analysis. To determine whether a trajectory is successful, we measured the distances between atoms located at the extremity of the domain IV of EF-G (residues 495–510, 525–545, and 570–580) and atoms located in the vicinity of the ribosomal protein L11. Using these criteria we collected two additional sets of 250,000 BD trajectories at two ionic strengths corresponding to 150 and 300 m*M* concentrations of NaCl. Figure 5 shows some of the encounter complexes (final positions of EF-G on the ribosome from successful trajectories) observed during simulations. The rate constant obtained at 150 m*M* ionic strength varies between 5 × 10^**7**^ and 1.2 × 10^8^ M^−1^ s^−1^, depending on the reaction distance criteria used that agrees with experimental values (see Introduction). The rate constant at 300 m*M* ionic strength is only about two times greater than the rate computed at 150 m*M* ionic strength, therefore, we concluded that the influence of bulk ionic strength on the rate of association is not significant.

## DISCUSSION

When this work was ongoing, the structure of the 70S ribosome from *T. thermophilus* complexed with EF-G was published (PDB codes 2WRI, 2WRJ^5^). This structure represents the post-translocational state of the 70S ribosome/EF-G complex. The conformation of the EF-G protein observed in the post-translocational state significantly differs from the one observed in the 1FNM crystal structure^29^ (Figure 1B). Particularly, the tip of the domain IV of EF-G is displaced about 30 Å. Flexible fitting of the EF-G structure to maps from electron microscopy have shown that EF-G undergoes large conformational change upon complexation with the ribosome.^59^ Because our aim was to model the initial stage of EF-G binding, we used the crystal structure of the isolated EF-G. We note, however that according to our recent molecular dynamics (MD) study,^39^ the EF-G protein is indeed flexible in solution and even though the conformational changes observed in the simulations were not as pronounced as seen in the post-translational complex^5^ (Figure 1B), they were still considerable.

As shown by normal mode analysis,^15–17^ single molecule experiments,^11^, ^13^, ^42^, ^60^, ^61^ cryo-EM,^62–65^ and crystallography,^3^ the translating ribosome is a highly dynamic assembly fluctuating between various conformational substates. A consequence of the two-subunit organization of the ribosome is the relative, ratchet-like movement of ribosomal subunits accompanied by both large and small scale structural rearrangements within individual subunits.^20^ This ratchet-like movement is required for translocation.^66^ According to our BD simulations, EF-G is not able to reach the A-site. It is possible that the internal, rachet-like movement of the ribosome may result in conformations in which the binding cleft would be more accessible for EF-G. Preferable positions of EF-G around the ribosome are these close to ribosomal protein L11 (Figures 3, 4, and 5). It seems that in the structure of the 70S complex employed in our work, the L11 protein is a major “obstacle” for EF-G on its way to the binding site. The L11 protein is a part of the L7/L12 stalk (GTPase-associated center), which is a highly dynamic fragment of the 50S ribosomal subunit.^20^ The base of this stalk is formed by the L10 protein bound to two dimers of L7/L12.^67^ The analysis of crystallographic structures of vacant ribosomes^2^ and cryo-EM reconstructions of EF-G bound ribosomes^41^, ^68^ reveal significant movements of the L11 stalk toward the A-site. According to our study, a rearrangement of the L11 protein (possibly in concert with a conformational change in EF-G) is required in order for the EF-G domain IV to reach the A-site.

The association rate constants of EF-G/ribosome complex obtained from our BD simulations can be treated only as a rough estimate, even though the agreement with the experimental values is reasonable.^1^ Although the BD simulations helped localize and define the encounter complex, an a priori knowledge of the orientation of EF-G and its contacts with the ribosome is needed to estimate the association rate constants with higher accuracy. We believe that other factors, as the lack of more sophisticated description of interactions including, for example, polarization terms^69^ and hydrophobic interactions,^70^ even though important, are secondary to this problem.

An interesting result of our BD study is that the EF-G/70S ribosome association rate constant only slightly depends on the bulk ionic strength. This effect can be explained by comparing the sizes of both the ribosome and EF-G with the effective range of electrostatic interactions in ionic solutions—between 150 and 300 m*M* the Debye length varies from 7.85 to 5.55 Å. As both molecules are negatively charged, the stronger screening observed at higher ionic strengths actually aids their association even though this effect is not pronounced. Apart from the monovalent bulk ionic strength accounted for in the Poisson-Boltzmann model, all Mg^2+^ ions from crystallographic structures of ribosomal subunits were kept at their original positions. The reason for including and representing Mg^2+^ ions explicitly is the following. Metal ions play an important role in folding and stabilizing RNA and RNA can form specific, negatively charged pockets to accommodate divalent cations.^71–75^ Here, we use Poisson-Boltzmann calculations to account for electrostatic interactions and this approach assumes that point-like ions are arranged according to the Boltzmann distribution. However, in cases where the electrostatic potential is significant (such as for highly charged ribosome), the assumption of Boltzmann distributed ions becomes inaccurate because it ignores the finite size and interactions of ions. As a consequence, an artificially high density of cations or anions is observed close to positively or negatively charged sites of a molecule (the shortcomings of the Poisson-Boltzmann theory in the treatment of ions are widely discussed in Refs. 76–80). A remedy for this problem is to either use continuum models that include finite sizes of ions^76^, ^81^ or to use explicit ions that are positioned at regions of large electrostatic potential. Appropriate positions of ions can be determined, for example, by using iterative procedures that recompute the electrostatic potential after adding a single explicit ion until no high electrostatic potential regions are present. However, for large and highly charged 70S ribosome, such procedure would be very expensive computationally or even not convergent, so instead of building positions of ions from the scratch we kept the positions of Mg^2+^ ions as in the crystallographic structure of the ribosome. Additionally, we note that theoretical methods used to predict positions of ions around nucleic acids are typically verified against crystallographic data.^82^, ^83^

## CONCLUSION

We performed BD simulations to estimate the most probable association paths of EF-G with the bacterial ribosome in its pretranslocational state. We observed high density areas of EF-G from the side of the L11 protein and the A-site, which suggest that this area is preferentially sampled by EF-G. However, there are also other regions of the ribosome surface sampled by EF-G, especially in the vicinity of positively charged ribosomal proteins. These observations suggest that there is no preferential, direct, electrostatic-driven path leading EF-G toward its binding site. The estimate of the EF-G/70S ribosome association rate at 150 m*M* ionic strength agrees reasonably well with experiments.

Our approach neglects the dynamic aspects of the EF-G/ribosome association. Both the ribosome^20^, ^84^ and EF-G^39^ are flexible and structural rearrangements are bound to play a role during their association. However, our conclusions on the lack of direct electrostatic steering should not be influenced by structural fluctuations of either EF-G or the ribosome. The estimates of association rates depend on the definition of the so-called transient (or encounter) complex. Based on our BD simulations we believe that for the EF-G/ribosome association the initial reaction state is located rather far from the final position of the EF-G at the A-site. We also conclude that the preferential position of the domain IV toward the A-site is necessary (Figures 2 and 5) for binding to occur. The question, as yet unanswered, is what happens after the initial encounter. The answer requires a methodology that is able to account for the flexibility of both molecules and cannot be answered by BD simulations.

Notwithstanding the limitations of the rigid-body BD approach and the Poisson-Boltzmann model, we believe that the screening of EF-G preferential areas and conformations around the ribosome can give a reasonable estimate of its association paths.

## MATERIALS AND METHODS

### Brownian Dynamics Method

The description of BD algorithms and their applications to estimate reaction rates of diffusion-controlled bimolecular reactions can be found elsewhere^48^, ^57^, ^85^; here we describe the BD method only briefly.

A BD trajectory is started by positioning the two randomly oriented molecules (the ligand and acceptor) at a distance *b* from each other. Next, the BD trajectory is propagated—both molecules (treated as rigid bodies described with translational and rotational diffusion coefficients of equivalent spheres) rotate and translate under the influence of stochastic forces originating from collisions with the surrounding solvent^47^, ^86^, ^87^ and deterministic intermolecular electrostatic interactions—until the molecules either react, in which case the trajectory is truncated, or their intermolecular separation reaches some predefined value *q*. When diffusing molecules reach the separation *q*, a decision is made based on a random number, whether the molecules have escaped from each other. The trajectory is either ended or the molecules are again placed at the relative distance *b* with orientations drawn from a specific analytically computed distribution and the trajectory is continued.^52^, ^85^ After a sufficiently large ensemble of BD trajectories is generated, the association rate constant is estimated based on the ratio of reactive versus escaped trajectories.^51^, ^52^

The electrostatic forces between diffusing solutes are evaluated based on precomputed solutions to the Poisson-Boltzmann equation^50^ that describes the electrostatic field generated by each (isolated) molecule. Coulombic forces acting on molecules can be computed by differentiating the potential energy of atom-centered point charges belonging to one molecule in the electrostatic field generated by the second molecule. The treatment of electrostatic interactions can be extended by including the effects of dielectric polarization at small intermolecular separations^69^ and nonpolar forces.^70^

### The Structure of the 70S Ribosome/EF-G Complex

The atomic structure of the 70S ribosome from *E. coli* along with associated structural magnesium ions used in this work was constructed based on data deposited in the Protein Data Bank under codes 3DF3 and 3DF4.^88^ All Mg^2+^ ions present in these two files were kept at their original positions.

The two tRNA molecules were positioned at A and P ribosomal sites based on the 1GIX structure.^89^ We used the 1FNM^29^ structure of the EF-G protein. We reverted the H573A point mutation and replaced the GDP molecule with GTP. Partial charges and atomic radii were assigned to structures using AMBER 9^90^ according to the Cornell et al. (1995) force field.^91^

To perform BD simulations with the aim of estimating the association kinetics, one needs to define reaction criteria that determine whether a particular BD trajectory leads to the reaction. Such criteria define the so-called transient complex ensemble,^92^ a region in the conformational space where the separations and orientations of associating molecules are close to those observed in their final bound complex. Properly defined reaction criteria represent relative conformations of molecules that are as close to the final complex as possible without the need to consider short-range interactions and conformational rearrangements. The transient-complex ensemble separates the bound and unbound states of molecules. Although in case of protein–protein association the reaction criteria are typically straightforward, based on crystallographic structures of complexes and tuned versus experimental data,^48^, ^53^, ^93–95^ it is not easy to define the reaction-criteria in the ribosome system. A major difficulty is that the ribosome is highly flexible^20^, ^84^ and characterizing the dynamical states of the ribosome with and without EF-G is, despite a lot of effort,^42^, ^61^, ^96–98^ still to be completed. We took the following approach to create the model of the initial 70S ribosome/EF-G complex. First, we extracted nucleic fragments and the EF-G molecule from the cryo-EM reconstruction deposited in the Protein Data Bank under the code 2OM7^99^ and superimposed them on the corresponding nucleic fragments of the 70S complex created from files 3DF3 and 3DF4 so that the root mean square deviation (RMSD) between phosphorous atoms was minimal. Next, the 1FNM EF-G structure was superimposed on the 2OM7 EF-G structure (with coordinates transformed in the previous step) using the RMSD of C_α_ atoms. These two operations resulted in the 1FNM EF-G structure positioned on the 70S ribosome. Next, we minimized the number of steric conflicts between EF-G and the ribosome. EF-G was translated and slightly rotated around its geometric center till the minimal distance observed within any pair of ribosome/EF-G atoms was not smaller than 2 Å. We then minimized the resulting structure using the sander module of AMBER and the GB^HCT^^100^ mezoscopic solvation model. Only the side chains of amino and nucleic acids were allowed to move during minimization. We did not allow for larger rearrangements of the EF-G and the ribosome as our intention was to build a model of the initial, or according to the philosophy described above, a transient 70S/EF-G complex. All structural positioning described above (except for minimization) was performed using a home-made software. Figure 2 shows the structure of the 70S ribosome/EF-G complex used in our BD simulations. Drawings of molecular structures were done using UCSF Chimera^101^ and VMD.^102^

### Visualization of the EF-G Association Pathway

Density maps represent the preferred positions of EF-G around the 70S ribosome. They were constructed based on a number of randomly chosen BD trajectories as follows. The positions of all EF-G atoms, taken relative to the central ribosome molecule from each BD trajectory, were transcribed (with a home-made software) into points belonging to regular cubic grids, with dimensions of 385 × 385× 385 and a 1.0 Å spacing, enclosing the ribosome. The resulting grids were then summed and normalized. The final density map was smoothed by convolution with a Gaussian function. Maps were visualized and analyzed with the UCSF Chimera package.^101^

### Electrostatic Calculations

Electrostatic potentials of the 70S ribosome at ionic strengths corresponding to 150 and 300 m*M* concentrations of NaCl were generated on three-dimensional Cartesian grids by solving the nonlinear Poisson-Boltzmann equation with the APBS package.^103^ The size of grids was 385 × 385 × 385, with a spacing of 0.9 Å in each direction. The dielectric constant of the ribosome was set to 4 and that of the solvent to 78.54. The van der Waals surface of the ribosome was used to define boundary between dielectric media. The temperature was set to 298.15 K.

The electrostatic potentials of the EF-G protein were generated by solving the linear Poisson-Boltzmann equation. In this case, grids of 289 × 289 × 289 size were used with a final spacing of 0.52 Å. The computed EF-G potentials served to derive effective charges^69^ used to represent the protein at 150 and 300 m*M* ionic strengths. To compute these effective charge, the PDC software (http://bionano. icm.edu.pl/software) was written. The effective charges are derived by fitting the electrostatic potential resulting from the Debye–Hückel approximation^104^ to the external molecular potential obtained as a numerical solution of the Poisson-Boltzmann equation. Fitting is performed by solving a linear system of equations^69^:

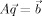
1

with the *A* matrix defined as:

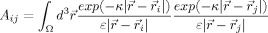
2

and the 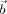
vector with coordinates given by:

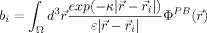
3

In the above equations, Ω denotes the volume outside a molecule where the electrostatic potential is fitted, 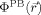
is the value of the electrostatic potential at a given point 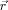
obtained by numerically solving the Poisson-Boltzmann equation, ε is the dielectric constant of the solvent, κ is the inverse of the Debye length and 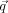
is the vector of effective charges. The accuracy (χ) of the obtained fit is defined as:

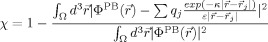
4

Potentials were fitted in a 6-Å-thick skin around EF-G with lower boundary of the skin defined as the van der Waals surface of EF-G inflated by 3 Å. Fitting errors were below 1%. The number of effective charges used to describe EF-G was 538 (the total number of EF-G atoms is 10,860). Effective charges were centered on EF-G polar atoms. We have also tried to derive effective charges by fitting the electrostatic potential resulting from the nonlinear version of the Poisson-Boltzmann equation, but we were not able to obtain satisfying accuracy. This is to be expected because the Debye–Hückel approximation is not valid in case of the nonlinear Poisson-Boltzmann equation in the vicinity of the electrostatic field source^105^ and much better agreement is obtained in the far field.

During BD simulations, the electrostatic energies and thus forces are evaluated using the values of the electrostatic potential (from the solution of the Poisson-Boltzmann on a three-dimensional grid) generated by the ribosome at positions of effective charges of the EF-G molecule.

### Simulations with Browndye

We used Browndye^85^ to simulate diffusional encounter of EF-G and the 70S ribosome. The *b* and *q* radii were set to 244 and 268.5 Å, respectively. The average minimum distance moved during a time step was set to 0.1 Å, to assure that forces are approximately constant during a particular step of the simulation. The positions of diffusing molecules were written to trajectory files every 100 steps. Only the long range electrostatic interactions were evaluated during simulations, based on potential grids computed with APBS and effective charges derived with PDC. Dielectric polarization effects^69^ were not included.
